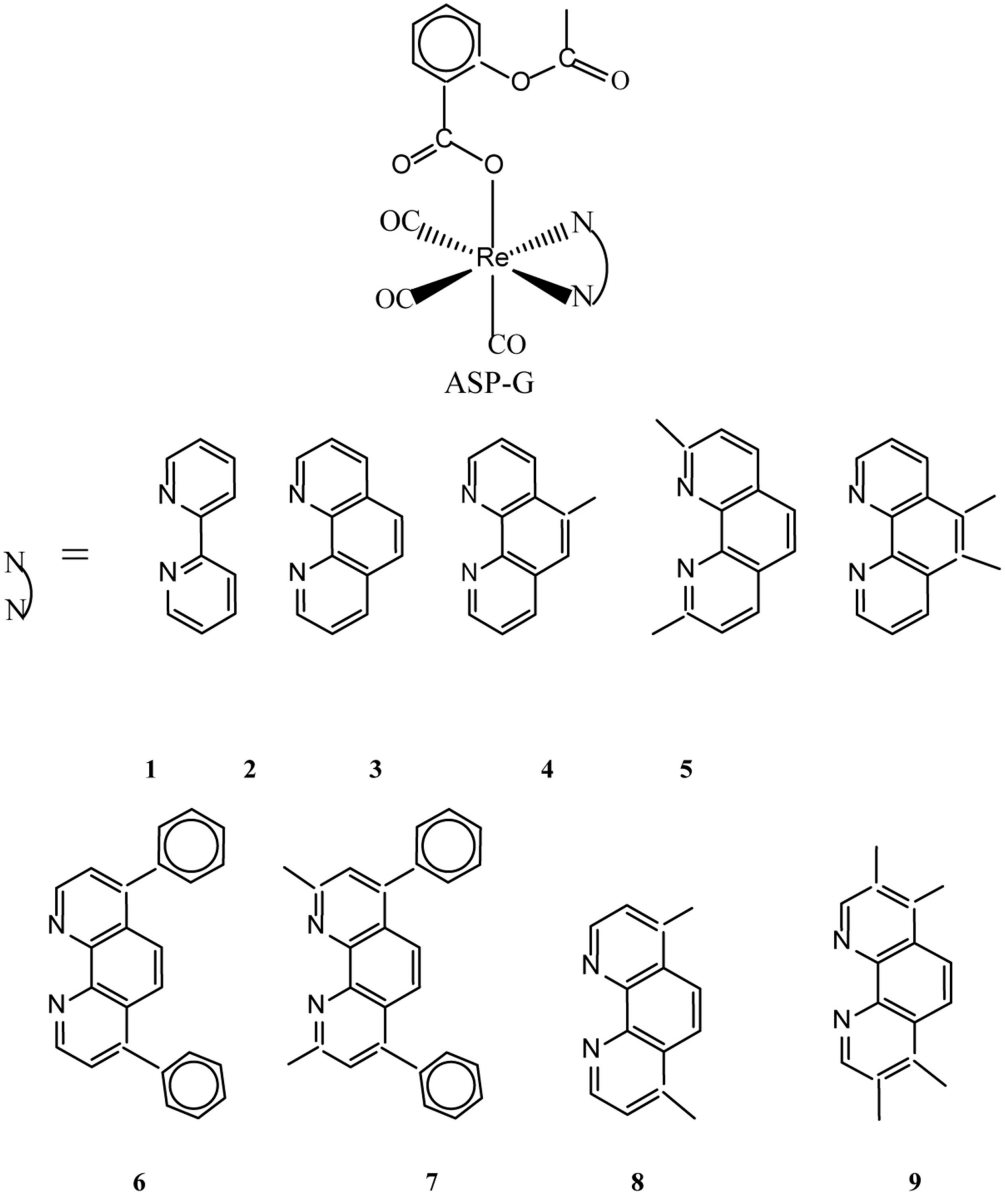# The Effects of Synthesized Rhenium Acetylsalicylate Compounds on Human Astrocytoma Cell Lines

**DOI:** 10.4172/1948-5956.1000512

**Published:** 2018-02-26

**Authors:** Hirendra N. Banerjee, Deidre Vaughan, Ava Boston, Gabriel Thorne, Gloria Payne, Josiah Sampson, Vinod Manglik, Pola Olczak, Brent V. Powell, Angela Winstead, Roosevelt Shaw, Santosh K Mandal

**Affiliations:** 1 Department of Natural, Pharmacy and Health Sciences, Elizabeth City State University, University of North Carolina, Elizabeth City, NC, USA; 2 Department of Chemistry, Morgan State University, Baltimore, MD, USA

**Keywords:** Astrocytoma, Rhenium, Cytotoxicity, DNA-binding

## Abstract

**Purpose:**

Because of the scarcity of suitable brain cancer drugs, researchers are frantically trying to discover novel and highly potent drugs free of side effects and drug-resistance. Rhenium compounds are known to be nontoxic and exhibit no drug resistance. For that reason, we have developed a series of novel rhenium acetylsalicylato (RAC or ASP) complexes to test their cytotoxicity on brain cancer cells. Also we have attempted to explore the DNAbinding properties of these compounds because many drugs either directly or indirectly bind to DNA.

**Methods:**

We have treated the RAC series compounds on human astrocytoma brain cancer cell lines and rat normal brain astrocyte cells and determined the efficacy of these complexes through in vitro cytotoxicity assay. We carried out the DNA-binding study through UV titrations of a RAC compound with DNA. Also we attempted to determine the planarity of the polypyridyl ligands of the RAC series compounds using DFT calculations.

**Results:**

RAC6 is more potent than any other RAC series compounds on HTB-12 human astrocytoma cancer cells as well as on Glioblastoma Multiforme D54 cell lines. In fact, The IC-50 value of RAC6 on HTB-12 cancer cells is approximately 2 μM. As expected, the RAC series compounds were not active on normal cells. The DFT calculations on the RAC series compounds were done and suggest that the polypyridyl ligands in the complexes are planar. The UV-titrations of RAC9 with DNA were carried out. It suggests that RAC9 and possibly all RAC series compounds bind to minor grooves of the DNA.

**Conclusion:**

Because of the very low activity of RAC6 on normal cells and low lC_50_ value of on astrocytoma (HTB-12) cell lines, it is possible that RAC6 and its derivatives may potentially find application in the treatment of brain cancers. The DFT calculations and UV titrations suggest that RAC series compounds either bind to DNA intercalatively or minor grooves of the DNA or both. However, it is highly premature to make any definite statement in the absence of other techniques.

## Introduction

Brain cancer is the leading cause of cancer-related death in children and the third most common cause of cancer death in the age group of 15-39. Primary brain cancers make up less than 2% of all cancers. Glioblastoma (GB) is the most common brain cancer and is recognized as one of the deadliest cancers [1]. Treatment options include a combination of surgery, radiation and chemotherapy. Many of the currently available chemotherapeutic drugs are alkylating agents such as cisplatin, carboplatin, carmustine, lomustine and cyclophosphamide. Others include topoisomerase inhibitors such as etoposide and innotecan [2]. Despite the devastating prognosis, there have been only a few FDA approved drugs to treat brain cancers in the past 30 years [3]. Although fewer side effects are observed for brain cancer drugs, there are always risks involved for chemo drugs. For example, platinum drugs can cause kidney damage and hearing loss [4].

Organometallic rhenium compounds are strong anticancer agents. Sometimes the IC_50_ values of many organometallic rhenium compounds against numerous cancer cells have been found to be in nM ranges [5]. We and others have demonstrated that organometallic rhenium compounds are not active on normal cells [5-8]. Many rhenium compounds have been found to be highly active on cancer cells resistant to chemo drugs [7-11]. Recently Collery have found out that organometallic rhenium diseleno ether complex is active on breast cancer cells resistant to conventional breast cancer drugs [6,10].

The first organometallic rhenium compounds found to be active on brain cancer cells (glioma UM-86) were reported in early 2000 [11-14]. These organometallic compounds include monometallic (**1**), dimetallic (**2, 3**) and trimetallic (**4**) octahedral rhenium complexes as shown in Scheme I. The ED_50_ values of the complexes are in the range 4.9 – 14.3 μg/mL. Although scores of similar organometallic rhenium complexes are now known [15], examples of the cytotoxic activity of these compounds on glioblastoma are scarce. We have recently synthesized a series of organometallic rhenium complexes (ASP-G) bearing the polypyridyl ligands, N^︵^N [5]. A scaffold for ASP-G is shown in Scheme II. For the compounds ASP1, ASP2, ASP3, ASP4, ASP5, ASP6, ASP7 and ASP8, the N^︵^N ligands are **1** (2,2′-bipridyl), **2** (1,10-phenanthroline), **3** (5-methyl-1,10-phenanthroline), **4** (2,9-dimethyl-1,10-phenanthroline), **5** (5,6-dimethyl-1,10-phenanthroline), **6** (4,7-diphenyl-1,10-phenanthroline), **7** (4,7-diphenyl-2,9-dimethyl-1.10-phenanthroline), **8** (4,7-dimethyl-1,10-phenanthroline), **9**(3,4,7,8-tetramethyl-1,10-phenanthroline) respectively. Here we present the cytotoxic properties of the ASP (aka RAC) series complexes against astrocytoma (HTB-12) and healthy astrocytes. Also included are the optimized structures of ASP1-ASP8 obtained from DFT calculations and UV-vis titration of ASP9 with DNA.

## Materials and Methods

### Synthesis of ASP series compounds

The compounds were synthesized through Mandal's synthesis [5]. In brief, a (1:1) molratio of the corresponding pentylcarbonato compounds [16] and acetylsalicylic acid were reacted in dichloromethane solvent to afford the corresponding RAC compounds.

### Cell Culture

Human astrocytoma (ATCC # HTB-12), rat astrocytes (ATCC # CRL-2005) cell lines were obtained (American Type Culture Collection, ATCC, Manassas, VA) D54 human Glioblastoma cell lines were kind gift from the laboratory of Dr. D. Bigner of Duke University, NC. HTB-12 cell lines were cultured in L-15 medium purchased from Caisson Labs, North Logan, UT with 10% fetal bovine serum and 100 μg/ml antibiotic/antimycotic (penicillin /streptomycin /amphotericin B). The cells were cultured in T 25-cm^2^ angle-necked Nunc flasks with filter caps and maintained in an incubator at 37°C. CRL-2005 Astrocytes cells were grown in T25-cm^2^ filter cap flasks which contained Dulbecco's Modified Eagle Medium (DMEM, Catalog # DML09) purchased from same company with 10% fetal bovine serum and 100 μg/ml antibiotic/antimycotic (penicillin /streptomycin /amphotericin B) and in a 5% CO_2_ incubator at 37°C,the D54 cell lines were cultured in a similar way but in DMEM/F12 medium obtained from the same company as described above.

### Drug Exposure Studies

Human astrocytoma cell lines HTB-12, rat astrocyte cell lines and D54 human GBM cells were plated in 6 well plates with 3mL of medium per well. When the cells were confluent, old medium was removed and 3mL of fresh medium was added containing different concentrations of compounds 1-7, which were previously dissolved in DMSO. Wells included negative control, DMSO vehicular control, and wells with desired concentrations of the drug. The cells were then placed back into their respectable incubators for a period 48 and 72 hours.

### Trypan Blue Assay

Cell death assay was done using Countess™ Automated Cell Counter by Invitrogen™ using the Trypan Blue dye exclusion method following standard protocol.

### LDH Assay

LDH release assay was done using a standard LDH assay kit from Clontech Corporation (USA), D54 GBM cell lines were exposed for 48 hours with 2 μm of RAC 6, the supernatant were collected and LDH release was measured using a standard microplate reader at 490 nm.

### DFT (density functional theory) calculations

We described it in our previous communication [17].

### UV-vis titration

The UV titrations of PC1 were reported previously [17]. The UV titrations of RAC9 were carried out in a similar manner. In brief, a DMSO solution of RAC9 diluted in Tris-HCl buffer saline into a cuvette was mixed gradually with an increasing amount of DNA and the spectrum was recorded after each addition of DNA.

## Results

**Figure 1:**
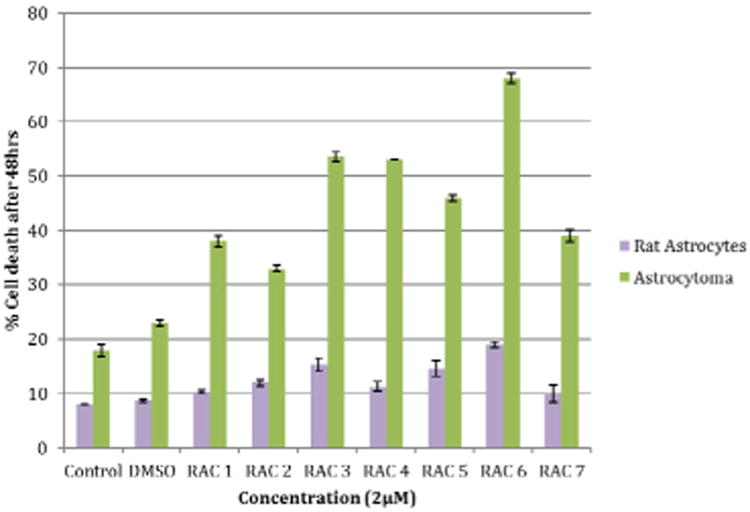
Cytotoxicity of Rhenium Acetylsalicylate Compounds (RAC1 - RAC7) on Astrocytes and Astrocytoma (HTB-12) cell lines at 48hrs. Rat astrocytes and astrocytoma cells were treated with a concentration of 2μM of RAC1 - RAC7. Cell count was determined using Countess™ Automated Cell Counter after 48hr incubation period. Bars are mean ± standard error (SE) of triplicate measurements. Rat astrocyte Control and DMSO measured at 8-9% cell death and RAC1 - RAC7 ranged from 10-19% cell death. Astrocytoma Control and DMSO measured at 18 and 23% cell death and RAC1 - RAC7 ranged from 33-68% cell death. The purpose of this graph was to look at the cytotoxicity of the RAC series compounds on rat astrocytes and astrocytoma cell lines after 48 hours.

**Figure 2:**
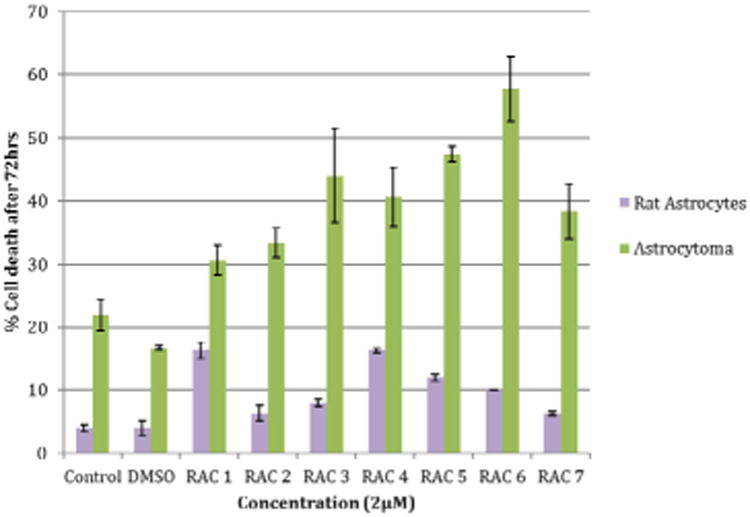
Cytotoxicity of Rhenium Acetylsalicylate Compounds RAC1 - RAC7 on Astrocytes and Astrocytoma (HTB-12) cell lines at 72hrs. Rat astrocytes and astrocytoma cells were treated with a concentration of 2μM of RAC1 - RAC7. Cell count was determined using Countess™ Automated Cell Counter after 72hr incubation period. Bars are mean ± standard error (SE) of triplicate measurements. Rat astrocyte Control and DMSO measured at 4% cell death and RAC1 - RAC7 ranged from 6-16% cell death. Astrocytoma Control and DMSO measured at 22 and 18% cell death and RAC1 - RAC7 ranged from 30-58% cell death. The purpose of this graph was to look at the cytotoxicity of RAC series compounds on rat astrocytes and astrocytoma cell lines after 72 hours.

**Figure 3:**
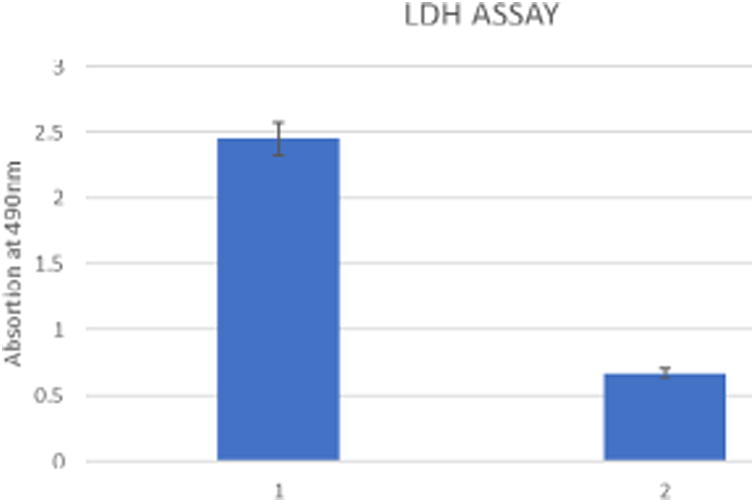
Effects of RAC6 on human Glioblastoma Multiforme D 54 cell lines after 48 hours of treatment; 1: RAC6; 2: DMSO treated.

**Figure 4:**
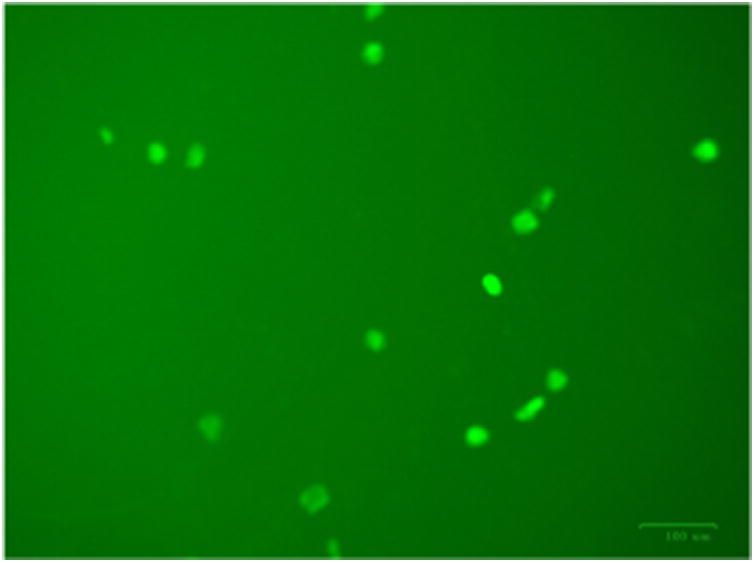
Annexin V-FITC stained RAC6 treated HTB12 cells showing increased apoptotic changes.

**Figure 5:**
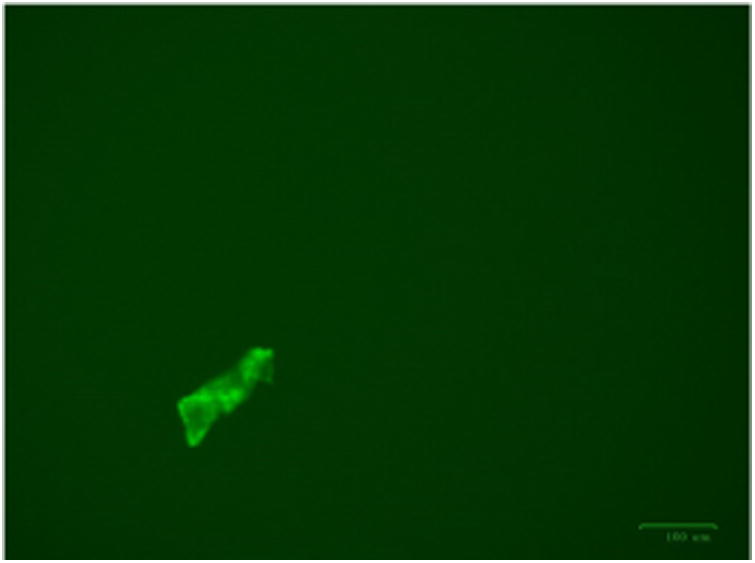
DMSO treated HTB12 cells showing almost no apoptotic changes.

Rat astrocytes and astrocytoma cells were treated with the RAC series compounds and the cell death assay was carried out using Trypan Blue Assay. Figure 1 shows the cytotoxicity of RAC1 – RAC7 on astrocytes and HTB-12 astrocytoma cell lines at 48hrs. Figure 2 exhibits the cytotoxicity of RAC1 – RAC7 on astrocytes and HTB-12 astrocytoma cell lines at 72 hours. Figure 3 shows the cytotoxicity of RAC6 on human Glioblastoma Multiforme D54 cell lines after 48 hours of treatment. The assay was carried out through LDH Assay. RAC6 also induced apoptosis in the treated cells as evidenced by increased green fluorescently labeled cells (hallmark of apoptotic functional assay) as compared to vehicular control (DMSO treated) cells (Figures 4, 5).

**Figure 6:**
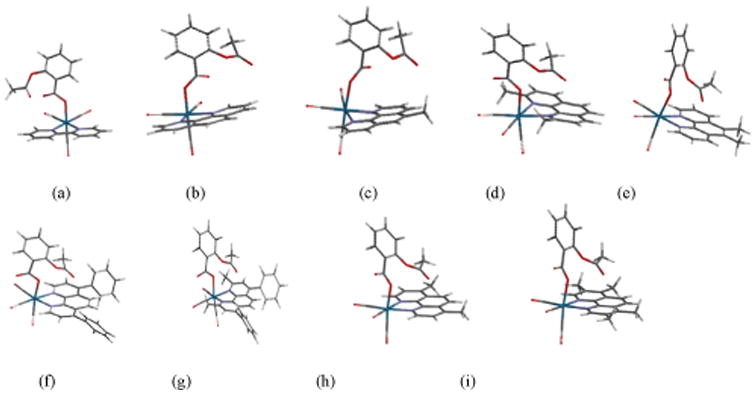
Optimized structures of ASP2-ASP9 obtained from DFT calculations showing the planarity of the phenanthroline rings of b-i (side view); a-i correspond to ASP1-ASP9, respectively.

The optimized structures of RAC2–RAC8 showing the planarity of 1,10-phenanthroline rings of the polypyridine ligands were determined by DFT calculations (Figure 6 b–g). The DFT B3LYP 6-31G(d) energies of HOMO and LUMO and the energy gaps in RAC1 – RAC9 are given as Supplementary Information.

**Figure 7:**
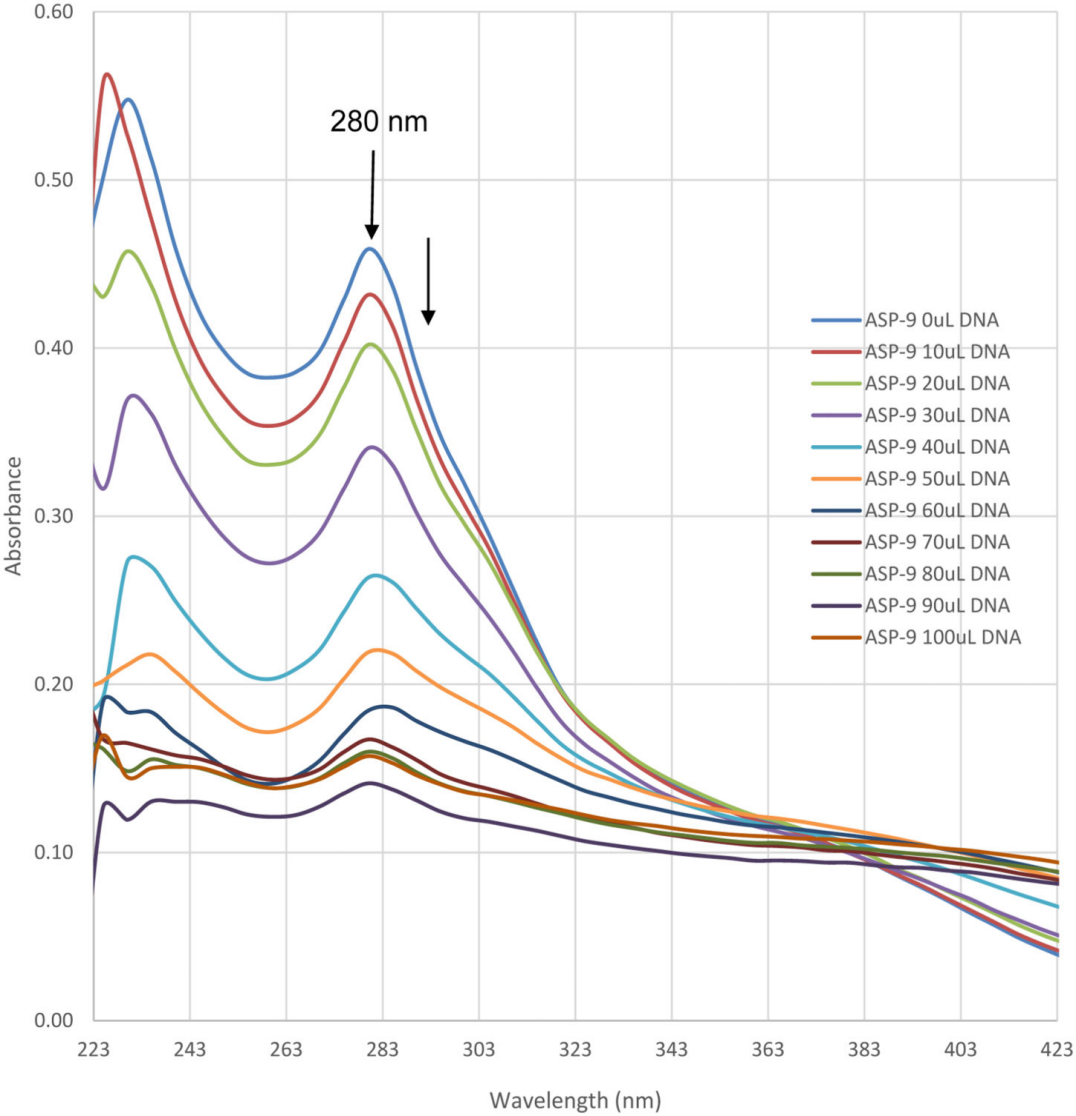
Electronic absorption spectra for the titration of 15 μM of ASP9 in the absence and presence of varied amount of DNA

The UV-vis spectra of RAC9 in the absence and presence of CT-DNA are shown in Figure 7 which indicates that the absorbance decreases with each addition of CT-DNA.

## Discussion

The RAC series compounds are a new class of anticancer agents. They have been found to be active against MCF-7 [5] and MDA-MB-231 [5] breast cancer cells, BxPC-3 pancreatic cancer [18], chemo resistant CR-HCT-116 and CR-HCT-29 colon cancer cells [19]. In the present study, RAC6 is more active than any other RAC-series compounds on HTB-12 human astrocytoma cancer cells (Figures 1, 2). RAC6 is much stronger than any compounds noted in Scheme I. The most effective compound is the dimetallic complex **3** shown in Scheme I. ED_50_ of this compound was reported to be 4.89 μg/mL which amounts to be 6.7 μM. The concentration of each RAC compound was 2 μM and roughly the IC_50_ value of RAC6 on HTB-12 cancer cells is ∼ 2 μM. Additionally we studied the cytotoxicity of RAC6 on human Glioblastoma Multiforme D54 cell lines (Figure 3). As expected, RAC6 is very active on this cell line too. Also Figures 1 and 2 suggest that RAC series compounds are not very active on normal cells. We have previously reported the cytotoxicity studies of IB series (Re-ibuprofenato) compounds. Both RAC and IB series compounds are derivatives of two NSAIDs (non-steroidal anti-inflammatory drugs). IB6 was very active on MCF-7 (IC_50_ ∼ 0.96 μM) and MDA-MB-231 (IC_50_ ∼ 1.1 μM) breast cancer cells. Structurally RAC6 and IB6 are very similar. Only the anionic ligands are different – one is acetylsalicylate and the other is ibuprofanate. It is therefore not surprising that both RAC6 and IB6 are very strong anticancer agents. Recently Lippard lab at MIT reported that a Re(V) complex bearing the same ligand 6 showed a very low IC_50_ value of ∼ 0.48 μM on breast cancer cells. It seems RAC6 induces apoptosis (Figures 4, 5)


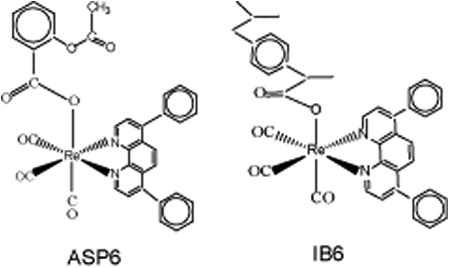


The phenanthroline rings in the RAC series compounds are planar as seen in the optimized structures of RAC2 through RAC9 (Figure 6). This indicates that the RAC series compounds RAC2-RAC9 may bind to DNA intercalatively. The UV titrations of RAC9 (Figure 7) show that the absorbance decreases with each addition of CT-DNA (hypochromic effect) without any red shift (bathochromic shift or shift to longer wavelength) Thus it is possible that RAC9 does not follow intercalation mechanism. It probably binds to the minor grooves of the DNA double helix which was observed in related rhenium complexes [20,21]. However, it is also possible that RAC9 partially binds to DNA intercalatively. In the absence of other techniques, it is hard to predict the actual binding mode.

## Conclusion

Because of RAC6's low IC_50_ value of ∼ 2 μM on astrocytoma (HTB-12) cell lines and very low activity on normal cells, RAC6 and its derivatives may potentially find application in the treatment of brain cancers.

## Supplementary Material

Suppl file

## Figures and Tables

**Scheme I:**
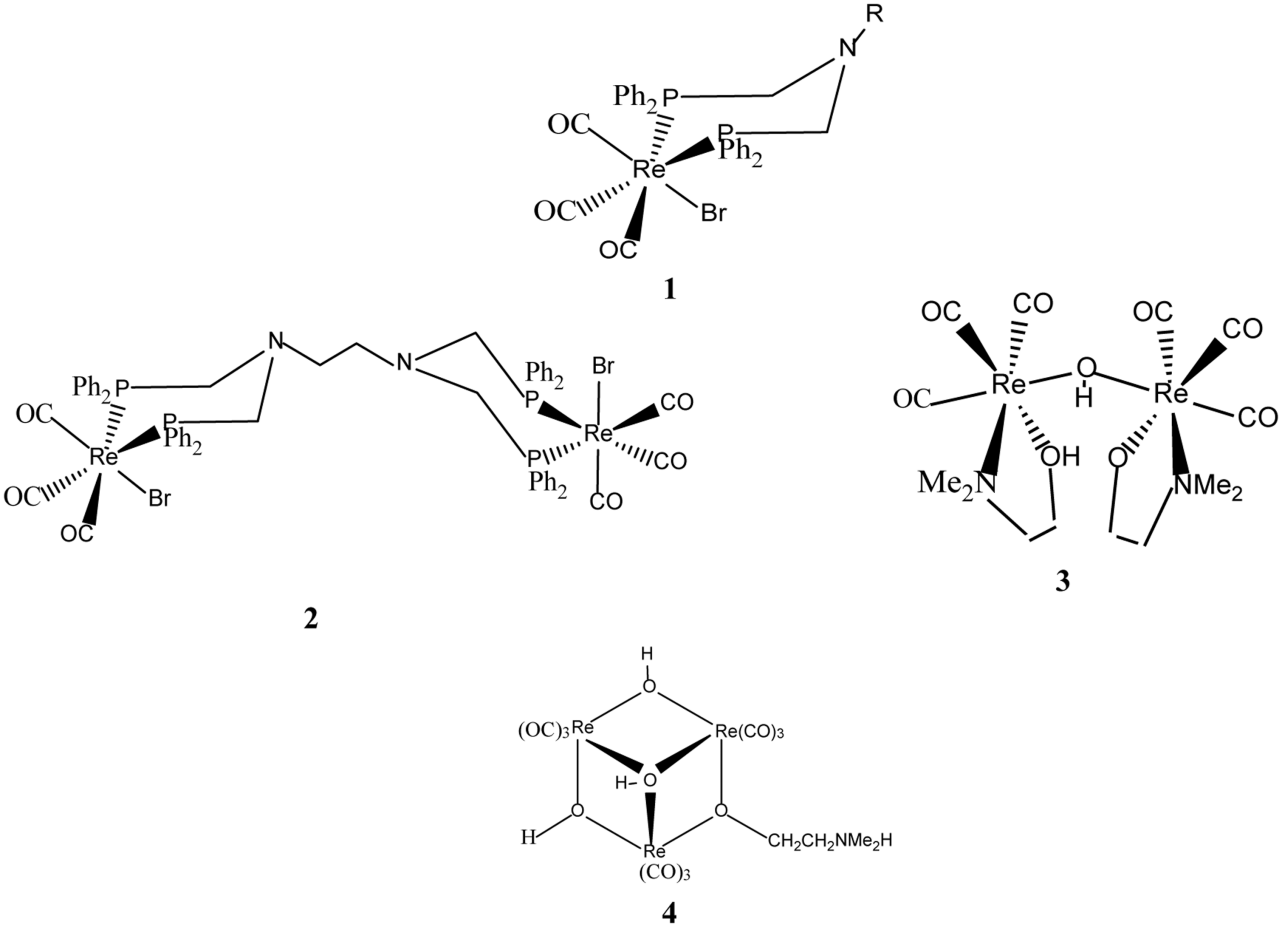


**Scheme II:**