# Supplementary material for: The Effects of Synthesized Rhenium Acetylsalicylate Compounds on Human Astrocytoma Cell Lines
Source: J Cancer Sci Ther. Author manuscript; Available in PMC 2018 Apr 24. (PMC5915335; doi:10.4172/1948-5956.1000512)
Supplement: Suppl file [file NIHMS954594-supplement-Suppl_file.docx]

**Supplementary Information**

Below are Spartan ’16 software gas phase DFT B3LYP/ 6-31G(d) optimized structures of ASP 1 - ASP 9, showing both their Ball- and- Wire and Tube models, and the locations of HOMO and LUMO with their corresponding energies

ASP1


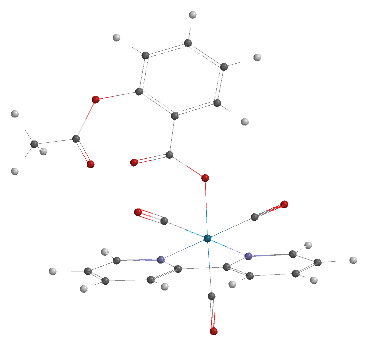

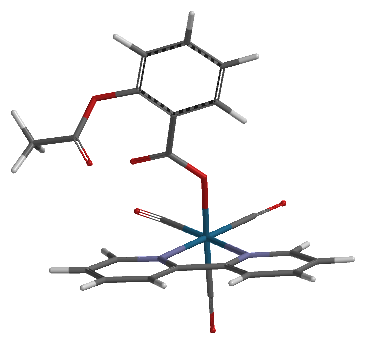


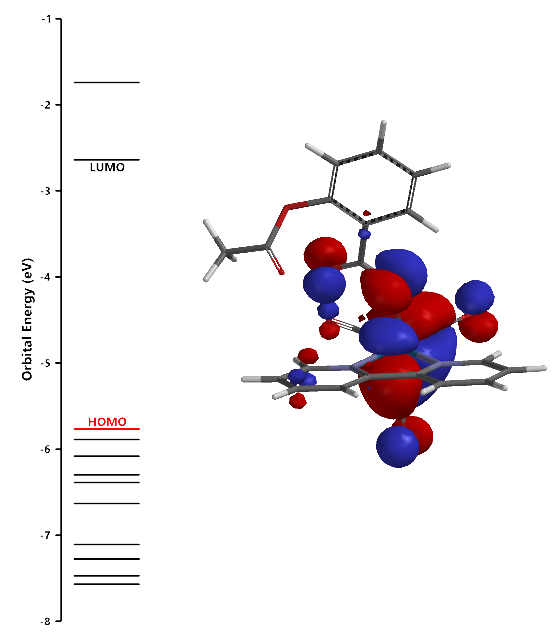

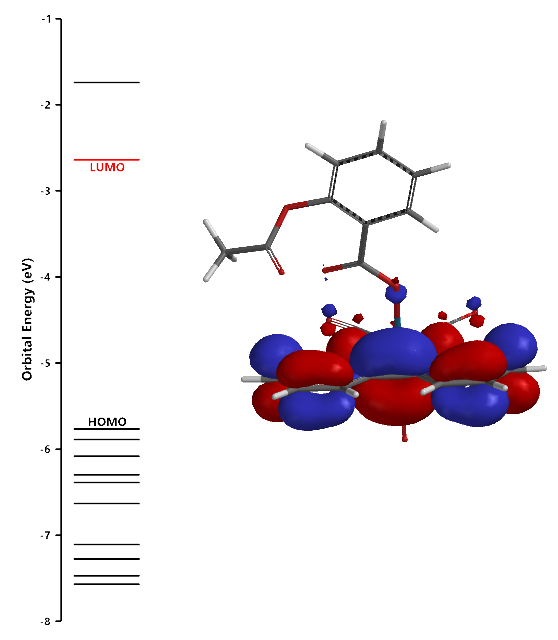


**EHOMO = -5.77 eV ELUMO =-2.64 eV**

**ASP-2**


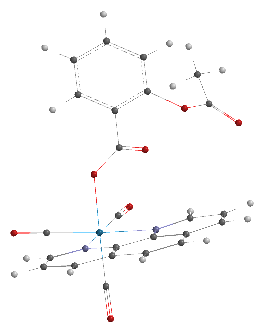

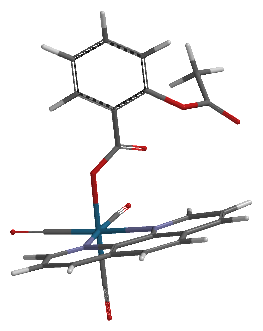

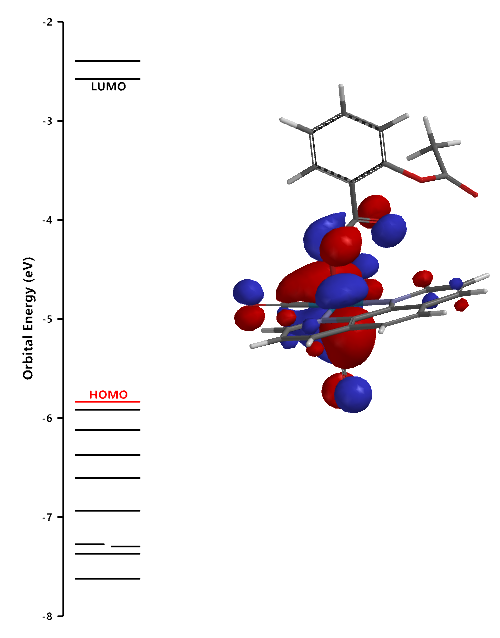

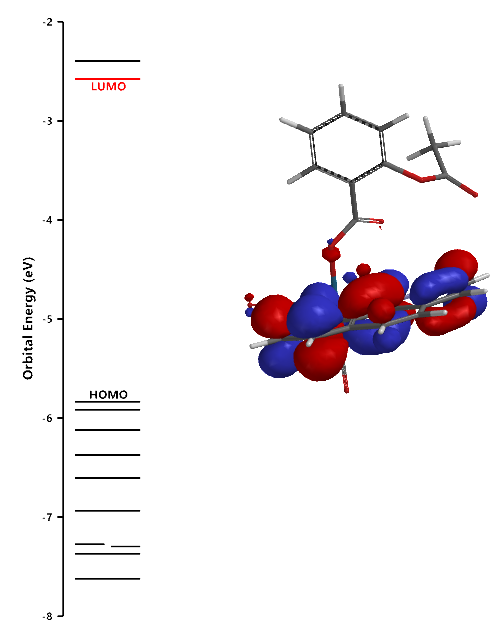


**E HOMO = -5.84 eV E LUMO = -2.58 eV**

**ASP-3**


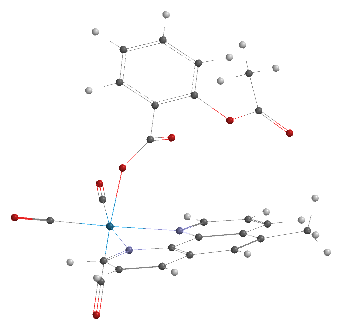

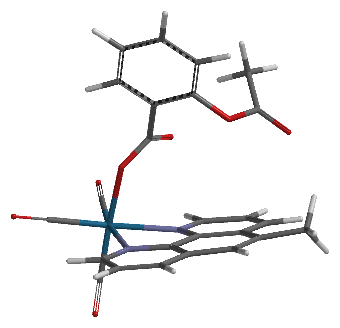


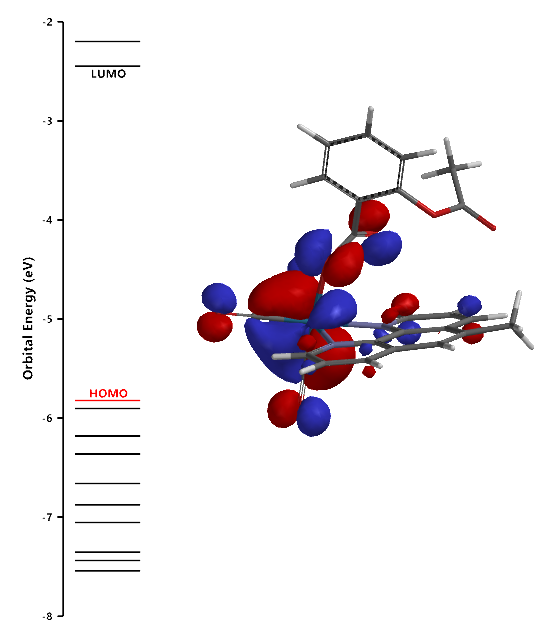

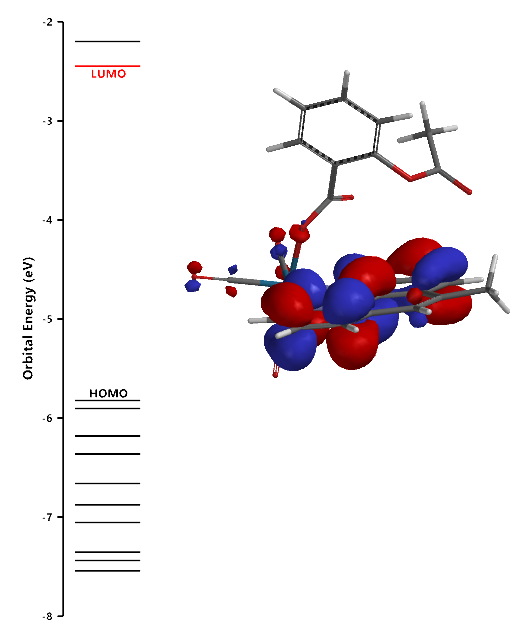


**E HOMO = -5.82 eV E LUMO = -2.45 eV**

**ASP-4**


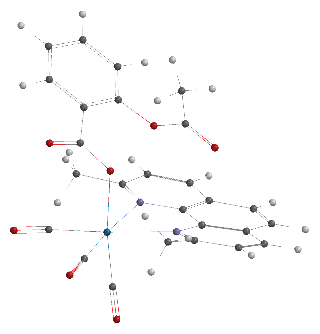

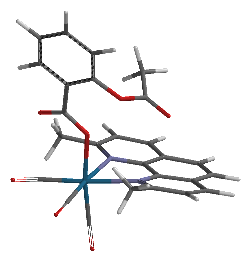


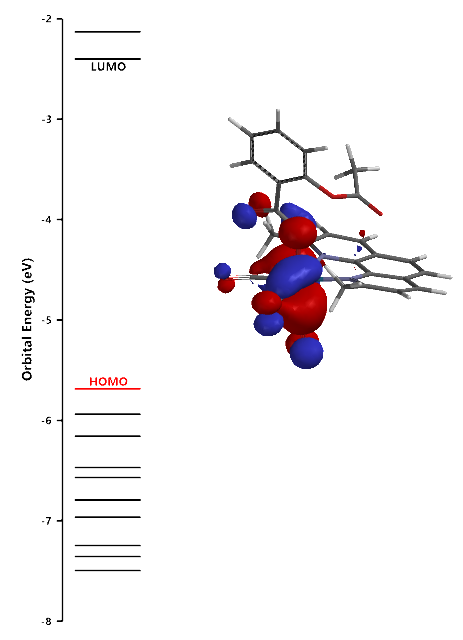

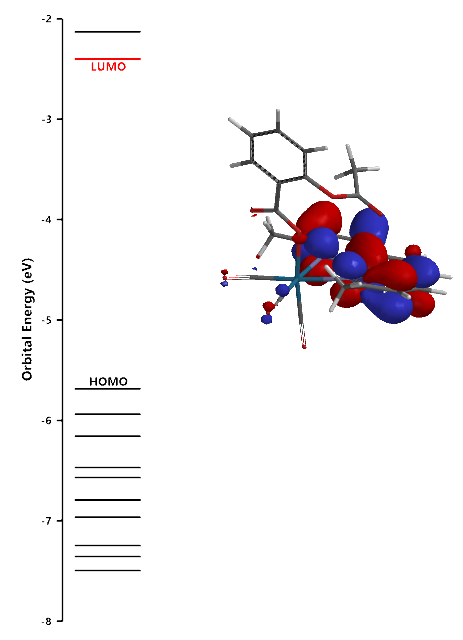


**E HOMO = -5.68 eV E LUMO = -2.40 eV**

**ASP-5**


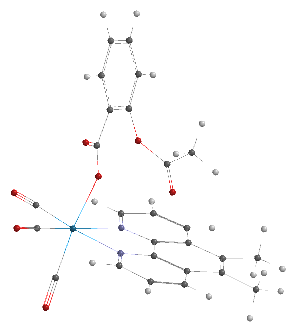

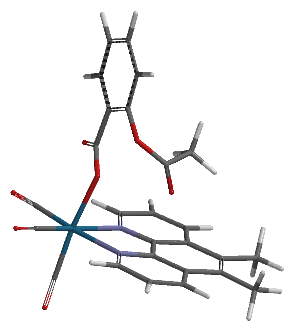


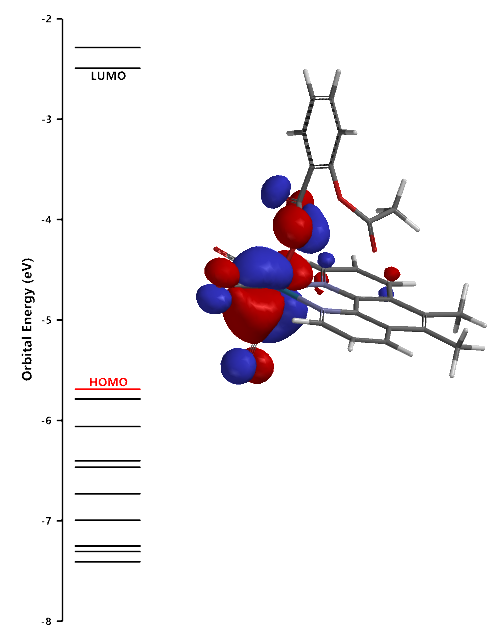

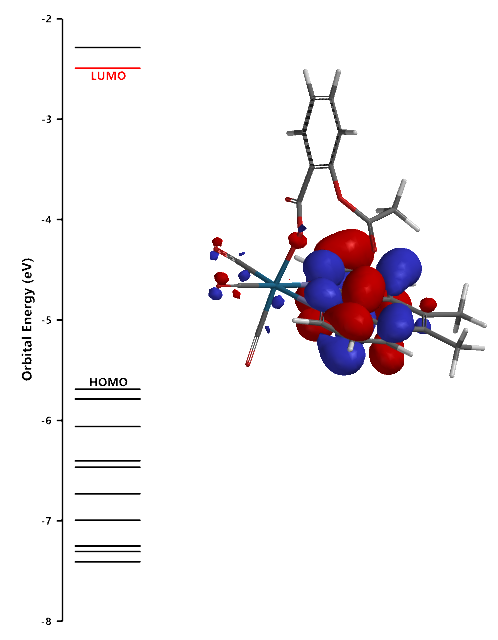


**E HOMO = -5.69 eV E LUMO = -2.49 eV**

**ASP-6**


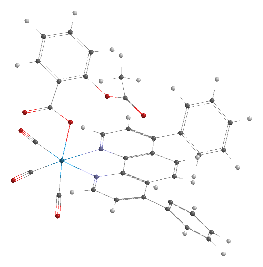

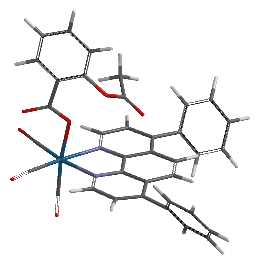


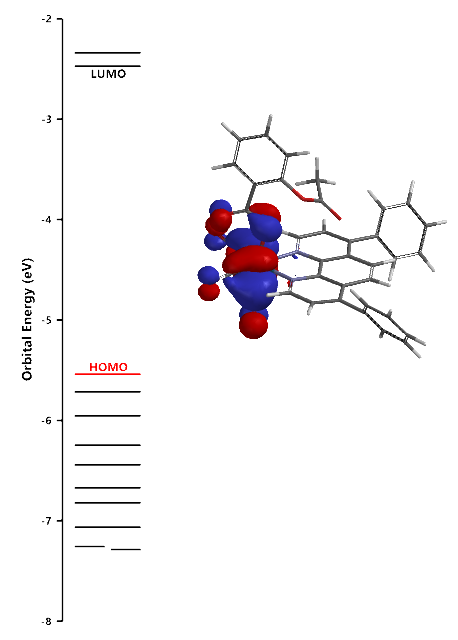

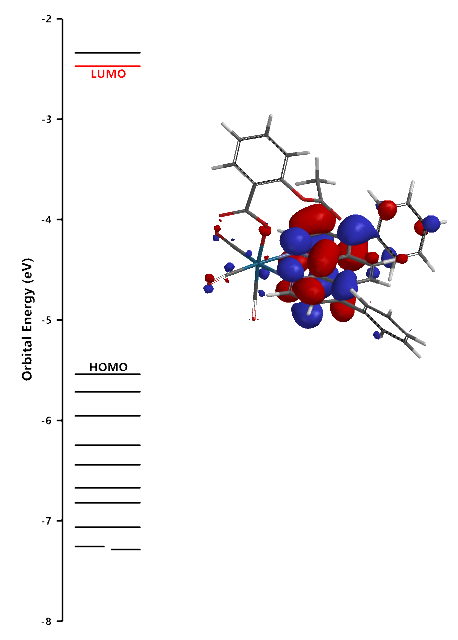


**E HOMO = -5.54 eV E LUMO = -2.47 eV**

**ASP-7**


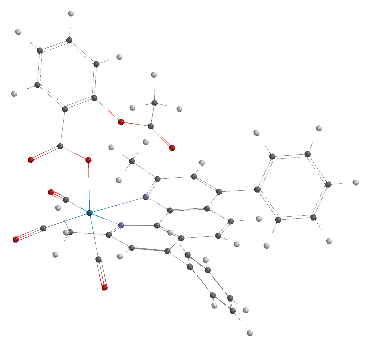

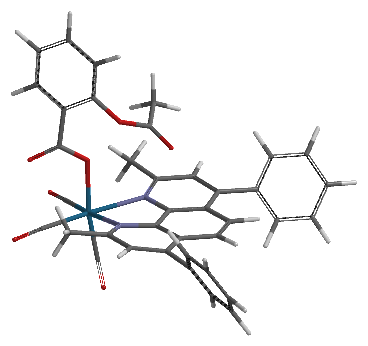


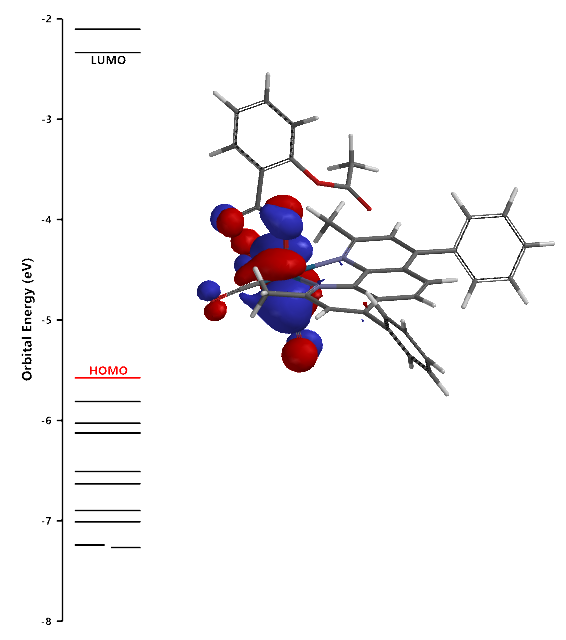

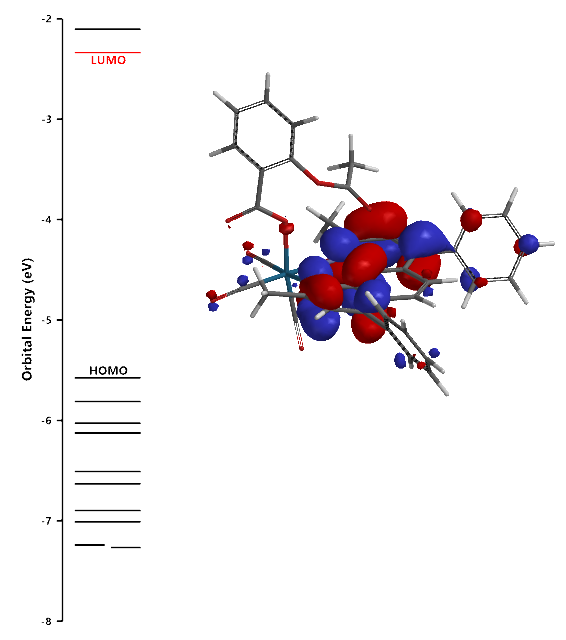


**E HOMO = -5.58 eV E LUMO = -2.34 eV**

**ASP-8**


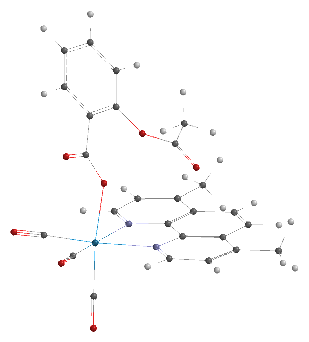

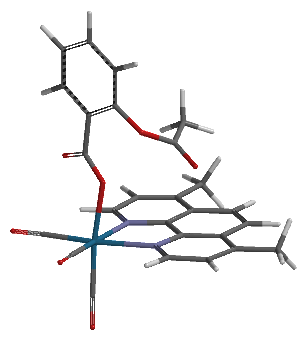


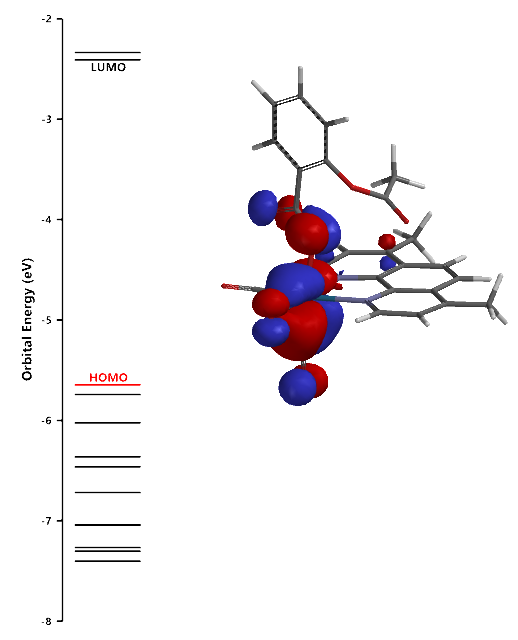

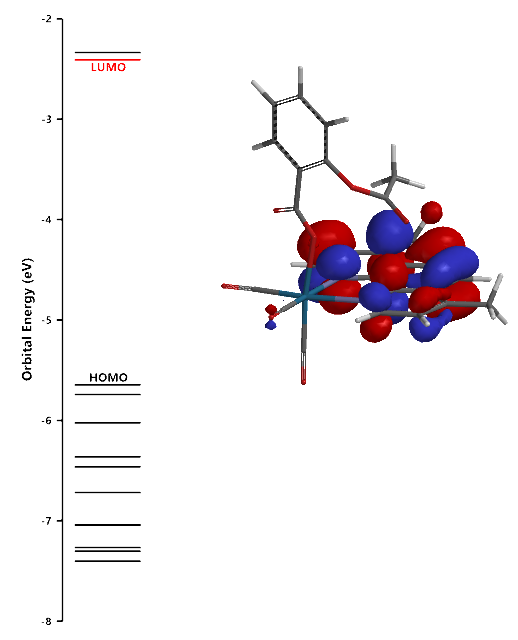


**E HOMO = -5.64 eV E LUMO = -2.41 eV**

**ASP-9**


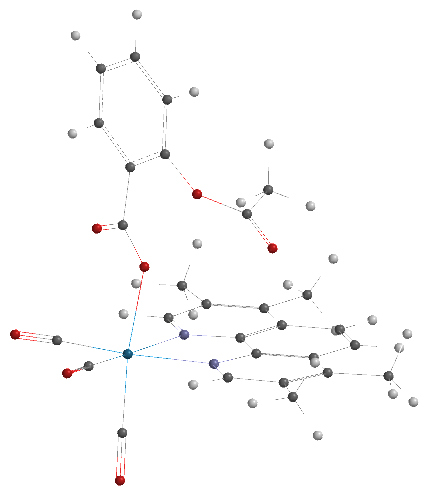

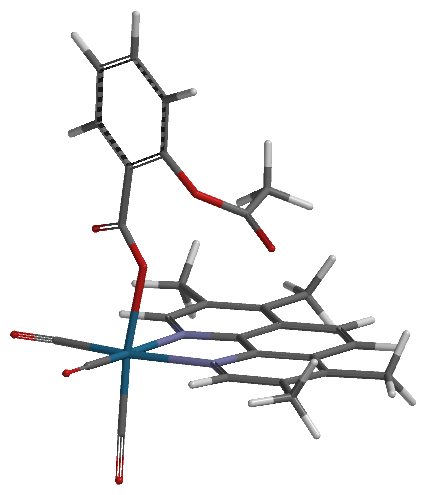


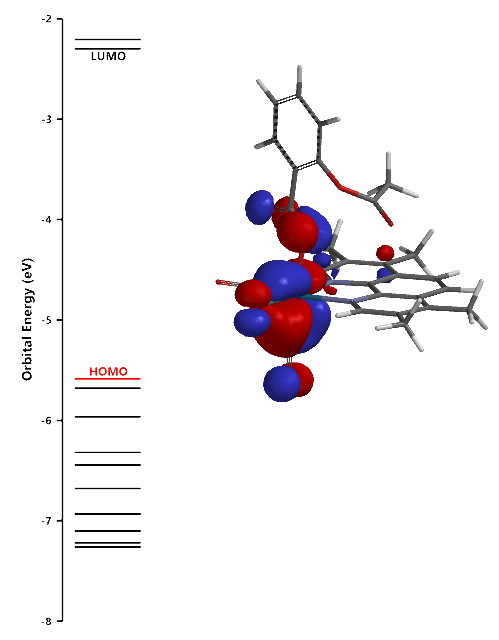

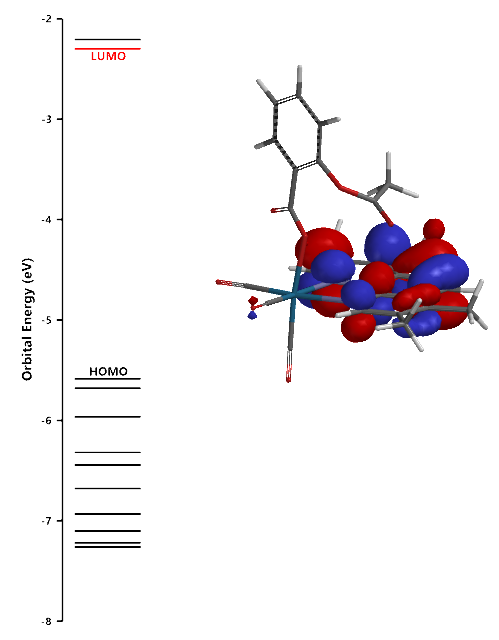


**E HOMO = -5.58 eV E LUMO = -2.30 eV**
